# Revisiting the effects of state earned income tax credits on infant health: a quasi-experimental study using contiguous border counties approach

**DOI:** 10.1186/s12889-023-17166-6

**Published:** 2023-12-05

**Authors:** Haobing Qian, George L. Wehby

**Affiliations:** 1 Department of Family and Community Medicine, University of California, San Francisco, USA; 2 Department of Health Management and Policy, University of Iowa, Iowa City, IA USA; 3 Department of Economics, University of Iowa, Iowa City, IA USA; 4 Preventive & Community Dentistry, University of Iowa, Iowa City, IA USA; 5 Public Policy Center, University of Iowa, Iowa City, IA USA; 6 National Bureau of Economic Research, Cambridge, MA USA

**Keywords:** Maternal and infant health, Earned income tax credit, Income inequalities, The earned income tax credits (EITC), Difference-in-difference (DID)

## Abstract

**Background:**

To examine the effects of refundable state earned income tax credits (EITC) on infant health.

**Methods:**

We use the restricted-access U.S. birth certificate data with county codes from 1989 to 2018. Birth outcomes include birth weight, low birth weight, gestational weeks, preterm birth, and the fetal growth rate. The analytical sample includes single mothers with high school education or less. Two specifications of two-way fixed effects models are employed. The first specification accounts for shared time trends across all states/counties. The second specification estimates effects based on EITC changes within contiguous counties across state borders which accounts for contemporaneous events specific to each contiguous county pair. Models are estimated pooling and stratifying by parity subgroups.

**Results:**

Under the first specification, refundable state EITC is associated with improved birth outcomes. Pooling all parity, a 10%-point increase in refundable EITC is associated with an 8-gram increase in birth weight (95% CI: 2.9,14.6). The effect increases by parity. In contrast, the estimates from the second model are much smaller and statistically non-significant, both pooling and stratifying by parity.

**Conclusions:**

Comparing contiguous counties across state borders, there is no evidence that refundable state EITC affects birth outcomes. However, the estimates still do not rule out moderate to large benefits for third or higher born infants.

**Supplementary Information:**

The online version contains supplementary material available at 10.1186/s12889-023-17166-6.

## Introduction

Family income is positively correlated with early infant health measures such as birth weight and gestational age in the United States [1–3]. However, whether changes in family income cause a change in infant health outcomes and the magnitude of such effects remains open questions. Some studies that have examined the effects of income-support policies in the US including the federal earned income tax credit or EITC, [4, 5] state EITC [6–8] and the minimum wage [9, 10] suggest an increase in birth weight and in some cases other related outcomes such as fetal growth or gestational age. There is however little evidence of a positive effect from the Aid to Families with Dependent Children program [11]. Studies examining other sources of variation in income (the Alaska permanent fund or parental job loss) also point to positive income effects on birth weight [12, 13]. Understanding the effects of income-support policies on infant health is especially important to evaluate the broader returns from such policies to population health.

In this paper, we revisit the evidence on the effects of state EITC on infant health. The evidence thus far has been mostly based on two-way fixed effects models that utilize variation in state EITC programs over time and across states, effectively comparing all states to each other. Two studies estimate this model using national birth certificate data from different periods and report an increase in birth weight. One study using data from 1980 to 2002 and whether the state has any EITC program reports that having an EITC program is associated with 18-gram increase in birth weight among single mothers of high school or less education [6]. The other study using data from 1994 to 2013 and examining whether EITC programs and refundable and are below 10% of the federal credit or not report an increase in birth weight from 9 g (non-refundable EITC, < 10% of federal credit) to 27 g (refundable EITC, ≥ 10% of federal credit) among mothers of high school or less education [7].

It is notable that the effect size from both studies is large, considering that these are intent-to-treat policy effects based on income effects from both recipients and non-recipients, and that state EITC is only a fraction of federal EITC. As we show below based on our own estimates from a similar model, these intent-to-treat policy estimates imply large and seemingly implausible income effects based on the overall evidence on income effects. Moreover, previous study with more recent data finds positive effects on birth weight for all four state groups with EITC (refundable/non-refundable, < or ≥ 10% of federal credit) for both single and married mothers, with some effects (including refundable and ≥ 10% of federal EITC) among married women exceeding those among single women [7]. This is rather unexpected a priori since the proportion of EITC recipients and average EITC amounts are lower among married than single mothers [14]. A key assumption from the TWFE model employed in these previous two studies is that contemporaneous events affecting the outcomes are shared across all states, including those that implemented EITC (irrespective of implementation time) and states that did not. This could be a strong assumption, however, considering that other economic changes may have occurred over time that differ between states, especially when comparing states that may differ substantially in their economic conditions and policies.

As an alternate to this model, we employ a model that compares contiguous counties across borders of states including those that differ in whether they have a refundable EITC and in the level of the credit. This model allows adds county pair by year fixed effects, which could isolate more of the change in infant health due to the differential change in the state EITC program across the contiguous cross-border county pair and remove potential confounding due to other contemporaneous changes in outcomes shared locally between contiguous border states than across pairs of states nationwide. This model is similar to that utilized in Dube et al. (2010) [15] to examine the effects of the minimum wage on labor market outcomes. In this study, we focus on refundable state programs which have been shown to have the largest association with infant health [7]and maternal health [14].

## Materials and methods

### Data and sample

The data comes from the U.S. Vital Statistic Natality Birth Data from the National Center for Health statistics (NCHS) [16]. It provides detailed information on the universe of live births occurring in the United States. We use the restricted-access Natality Data files from 1989 to 2018 which provides geographic information including state and county geocodes. The analytical sample for this study includes infants born to single mothers with an education of high school or less aged 18–46 at the time of delivery to focus on the sample that is most likely to be eligible (and therefore affected by EITC). We aggregate the data to the county level for each study year. This study was exempt from IRB review and data were analyzed in September 2022.

### Study measures

#### EITC measure

The primary independent variable, the EITC measure, is the refundable state EITC credit as a percentage of the federal credit. States set the credit level as a fixed percent of the federal level; therefore, this measure captures differences in EITC generosity across states and is analogous to using the maximum credit as the exposure measure. In the individual-level data, before aggregating the data for each county and year, the EITC measure is assigned to 0 for states with no EITC and states with nonrefundable EITC since we find little evidence of the health effects from non-refundable state EITC on maternal health [14] and there are only 6 states offering non-refundable EITC in tax year 2018. state credit levels ranged from 3.5 to 85% (California has 85% of federal but the income eligibility is not based on federal rules and has a narrow range). State EITC data are obtained from the National Bureau of Economic Research [17] and the Urban-Brookings Tax Policy Center [18].

#### Covariates

We include the following covariates aggregated to the county level from individual-level information: proportions of the sample by maternal age categories (18–24, 25–29, 30–34, 35–39, or 40–46 years), education level (high school or less), and race/ethnicity (non-Hispanic Whites, non-Hispanic Blacks, Hispanics, or other race/ethnicity) groups, and child’s sex. State-level contextual covariates include the real minimum wage, [19] two indicators for whether the state had implemented Aid to Families with Dependent Children (AFDC) or Temporary Assistance for Needy Families (TANF) in a given year, [20] and the Medicaid maximum income eligibility for pregnant women obtained from Dave et al. (2010) [21] and the Kaiser Family Foundation [22].

#### Outcome measures

We examine the following infant health outcomes all aggregated at the county level: (1) birth weight mean in grams; (2) proportion of low birth weight (less than 2500 g) infants; (3) mean gestational age in weeks; (4) proportion of preterm birth (gestational age < 37 weeks) infants; and (5) the mean fetal growth rate (birthweight/gestational age).

### Statistical analysis

#### All counties sample

We first estimate the effects using a general difference-in-difference model (a two two-way fixed effects model) including all counties with population of 100,000 or more (counties with population fewer than 100,000 are not identified in the data) [23]. Number of counties ranges from 1632 to 3113 over the study period. The model utilizes within-state variation comparing counties in states with changes in refundable EITC credits (including enacting a new program or modifying credit levels) to counties in states with no changes, while estimating and controlling for time-invariant differences between counties (and states) and national trends in outcomes shared across counties and states. The model is specified as follows using county-level aggregated data:

1 $${Y_{cst}} = {\alpha _0} + {\alpha _1}REFUND\_EIT{C_{cst}} + '{\alpha _3}{\gamma _{st}} + {\theta _c} + {\lambda _t} + {e_{st}}$$

Where Y_cst_ is one of the outcome measures for infants born in county c in state s in birth year t. REFUND_ EITC_s(t−m)_ is the refundable state EITC as the percent of federal EITC in tax year t-1 or t-2. Because the majority of EITC tax refunds are received in February, [24] we assign EITC parameters one calendar years ago EITC_s(t−1)_ for births occurring during the months of May to December (third trimester beginning from February to September) and assign EITC parameters two calendar years ago EITC_s(t−2)_ for births occurring during the months of January to April (third trimester beginning from October to January), assuming the immediate income effects of EITC on infant health spent within the subsequent 12 months upon receipt and based on evidence suggesting that the third trimester is critical for birth weight production [25, 26]. θ_c_ is county fixed effects, λ_t_ is year fixed effects, and γ_st_ are the state-level time-varying control described above. X_ct_ are county-level demographic characteristics including maternal age, race/ethnicity, education, and child sex aggregated from the individual-level data so that we have one observation per county per year. e_st_ includes the error term. We estimate the model using weighted least squares using the count of observations in the county-level summary outcome as the weight, and cluster the standard errors at the state level. Since EITC amounts differ by the number of children, with larger amounts for more children (capped at 2 or more until 2008 and then 3 or more beginning in 2009), we estimate the models pooling and stratifying by parity subgroups (1st, 2nd, and 3rd or higher). EITC amounts for childless adults (which are the amounts applicable to 1st born children) are small (for example, maximum federal credit of $529 in 2019). Because of this and state credits being a fraction of the federal credit, we expect changes in refundable state EITC to have little to no effect on 1st born children, which we evaluate empirically. Our model does not leverage differences in EITC resulting from parity differences to estimate the EITC effects. Rather, when we stratify by parity, we estimate he intent-to-treat policy effects resulting from changes in maximum state credit levels over time separately by parity.

#### Contiguous Border county-pairs sample

One concern with model (1) is that states and counties may have different time trends, in which case the shared timed trends might not adequately capture the contemporaneous events that possibly confound EITC changes and their effects, such as local economic trends. Therefore, we estimate another model based on contiguous counties across state borders to further account for local time-varying trends. Cross-border contiguous counties might share more of these contemporaneous events due to similarities in the local economy and cross-county economic and social interactions than distant counties and states. At the same time, because the EITC depends on state of residence, there is little concern about an effect from crossing the county border for work on the estimates. For this analysis, we restrict the sample to contiguous county-pairs sharing a state border included in the county adjacency file from the Census Bureau [27]. This sample consists of 1308 contiguous county-pairs, which give a panel of 40,548 county-by-year observations, with an annual observation for each county for each pair; a county would have two repeated observations in a given year if it shared a border with two counties in the neighboring state. The model is specified as follows:

2 $${Y_{cspt}} = {\alpha _0} + {\alpha _1}REFUND\_EIT{C_{cst}} + {\alpha _3}{X_{ct}} + {\alpha _4}{\gamma _{st}} + {\theta _c} + {\rho _{pt}} + {\varepsilon _{ist}}$$

In Model 2, p represents a cross-border county pair. The key distinction between models (1) and (2) is that model (2) replaces the year fixed effects with county-pair by year fixed effects ρ_pt_; in that way, the model utilizes within cross-border county-pair variations in EITC over time and removes time-varying confounders shared between contiguous counties. Similar to the previous model, we estimate this model with weighted least squares with standard errors clustered at the state of county c. We also estimate the models pooling and stratifying by parity. Finally, to check for whether differences in estimates between models (1) and (2) are due to the difference in included counties (model 1 is estimated for all counties, while model 2 only for contiguous counties) rather than the regression specification, we re-estimate model (1) only including border counties.

## Results

### Descriptive analysis

Figures S1 and Figure S2 show the proportion of county-pairs with a refundable EITC difference, and the average percent EITC difference, respectively, from tax year 1988 to 2017. Number of county-pairs that had a difference in refundable EITC increased substantially, indicating the variation in refundable EITC over time and between contiguous county-pairs.

Table S1 shows descriptive statistics for birth outcomes and demographic control variables for the analytical sample. The average birth weight is 3210 g and the low birth weight rate is 10%; the average gestational age is 38.7 weeks, and the preterm birth rate is 10%. About 27% of the sample are non-Hispanic Black, 39% are non-Hispanic White, 30% are Hispanic, and 4% are of other race/ethnicity.

### Estimates from the all-county sample

Table 1 shows the estimates model (1) pooling by birth order. We find that refundable EITC is associated with improved birth outcomes. Specifically, a 10%-point increase in refundable EITC is associated with an increase by 8 g in birth weight (p < 0.01), 0.05 weeks in gestation weeks (p < 0.01), and 1.1 g per 10 gestational weeks (p < 0.05), and a 0.3%-point decrease in the low birth weight rate (p < 0.01).

**Table 1:** Effects of a Ten-Percentage-Point Increase in Refundable State EITC (as % of Federal Credit) on Birth Outcomes Born to Single Low-Educated Women Aged 18–46 Years, Natality Files 1989–2018, All County Sample

Outcomes	EITC Effect (95% CI)	Outcome Mean
*All birth order*		
Birth weight (grams)	8.237**	3209.88
	[2.90,13.58]	
Low birth weight rate (%)	-0.003**	0.096
	[-0.005, -0.001]	
Gestational weeks (week)	0.052**	38.67
	[0.017,0.087]	
Preterm birth rate (%)	-0.003	0.14
	[-0.006,0.00008]	
Fetal growth rate (grams/week)	0.110*	82.79
	[0.015,0.21]	

Note: Each cell represents the effect of a 10%-point increase in refundable state EITCs (relative to federal credits) in tax year t-m on an outcome in birth year t. The EITC is lagged two years prior to birth year (t-2) for birth months during January to April and lagged one year prior to birth year (t-1) for birth months during May to December. Estimates are county level aggregated data analyses. Each outcome and demographic characteristics are the average value for each county per year (one observation for each county per year). The model includes one EITC variable, the refundable percentage of federal credit (states with no EITC and states with non-refundable ETIC have 0 on this variable as the control group). The demographic controls include indicators for maternal age, child sex, race/ethnicity, education, and county and year fixed effects. State level controls include average minimum wage (2018$) from the past 12 months, average state cigarette tax from the past 12 months, and average maximum Medicaid income eligibility for pregnant women as % of FPL from the past 12 months. The sample size is 240,346. The regressions are weighted using the count of outcomes at the county level. Standard errors (SE) are clustered at state level and shown in paratheses. ^*^p < 0.05, ^**^p < 0.01

When stratifying model (1) by birth order (Table 2), EITC effects are largest for third or higher born infants (whose mothers receive higher EITC credits for a given qualifying income). The estimates for firstborn infants (whose mothers receive little EITC credit on average) are noticeably smaller and statistically non-significant for birth weight (as expected) but are still noticeable for gestational age and low birth weight, indicating potential bias in this model.

**Table 2:** Effects of a Ten-Percentage-Point Increase in Refundable State EITC (as % of Federal Credit) on Birth Outcomes Born to Single Low-Educated Women Aged 18–46 Years, Natality Files 1989–2018, All County Sample by Birth Order

Outcomes	EITC Effect (95% CI)	Outcome Mean
*first order birth order*		
Birth weight (grams)	4.262	3196.27
	[-0.374,8.89]	
Low birth weight rate (%)	-0.002*	0.096
	[-0.003, -0.00009]	
Gestational weeks (week)	0.042*	38.90
	[0.007,0.078]	
Preterm birth rate (%)	-0.001	0.123
	[-0.004,0.001]	
Fetal growth rate (grams/week)	0.024	81.92
	[-0.05,0.10]	
*second birth order*		
Birth weight (grams)	7.823**	3226.15
	[2.87,12.77]	
Low birth weight rate (%)	-0.003**	0.089
	[-0.005, -0.001]	
Gestational weeks (week)	0.045**	38.67
	[0.014,0.077]	
Preterm birth rate (%)	-0.002	0.135
	[-0.005,0.001]	
Fetal growth rate (grams/week)	0.114*	83.25
	[0.02,0.21]	
*third or higher birth order*		
Birth weight (grams)	12.411**	3200.94
	[5.48,19.34]	
Low birth weight rate (%)	-0.005**	0.108
	[-0.008, -0.002]	
Gestational weeks (week)	0.061**	38.40
	[0.024,0.099]	
Preterm birth rate (%)	-0.005*	0.160
	[-0.009, -0.001]	
Fetal growth rate (grams/week)	0.201**	83.09
	[0.067,0.33]	

Note: Each cell represents the effect of a 10%-point increase in refundable state EITCs (relative to federal credits) in tax year t-m on an outcome in birth year t. The EITC measure, each outcome and demographic characteristics are the average value for each county per year (one observation for each county per year). The model includes one EITC variable, the refundable percentage of federal credit (states with no EITC and states with non-refundable ETIC have 0 on this variable as the control group). The demographic controls include indicators for maternal age, child sex, race/ethnicity, education, and county and year fixed effects. State level controls include average minimum wage (2018$) from the past 12 months, average state cigarette tax from the past 12 months, and average maximum Medicaid income eligibility for pregnant women as % of FPL from the past 12 months. The regressions are run separately for each birth order. The sample size is 81,589 for first birth order, 79,536 for second birth order, and 79,221 for third or higher birth order. The regressions are weighted using the count of outcomes at the county level. Standard errors (SE) are clustered at state level and shown in paratheses. ^*^p < 0.05, ^**^p < 0.01

### Estimates from the cross-border contiguous counties model

Table 3 shows the estimates from model (2) pooling across birth orders. Compared to the estimates from Model (1), Model (2) estimates are noticeably smaller – for example, the effect estimate for birth weight is only 9.2% of that from model (1) – and are statistically non-significant. Furthermore, even though the standard errors for birth weight and fetal growth are slightly larger (about 15% more for birth weight), the 95% confidence intervals (CIs) rule out the larger estimates for effects on birth weight and gestational age from model (1).

**Table 3:** Effects of a Ten-Percentage-Point Increase in Refundable State EITC (as % of Federal Credit) on Birth Outcomes Born to Single Low-Educated Women Aged 18–46 Years, Natality Files 1989–2018, Contiguous County Sample

Outcomes	EITC Effect (95% CI)	Outcome Mean
*All birth order*		
Birth weight (grams)	0.76	3198.72
	[-5.90,7.42]	
Low birth weight rate (%)	-0.001	0.10
	[-0.004,0.002]	
Gestational weeks (week)	0.010	38.70
	[-0.022,0.041]	
Preterm birth rate (%)	-0.001	0.14
	[-0.004,0.003]	
Fetal growth rate (grams/week)	-0.003	82.42
	[-0.13,0.13]	

Note: Each cell represents the effect of a 10%-point increase in refundable state EITCs (relative to federal credits) in tax year t-m on an outcome in birth year t in counties located in states with refundable EITC programs, compared to the border counties within each county-pair. The EITC measure, each outcome and demographic characteristics and state level control variables are the average value for each county per year. Each county may have multiple observations per year since a single county may have multiple contiguous county pairs. The model includes one EITC variable, the refundable percentage of federal credit (states with no EITC and states with non-refundable ETIC have 0 on this variable as the control group). The demographic controls include indicators for maternal age, child sex, race/ethnicity, education, and county-pair by year fixed effects. State level controls include average minimum wage (2018$) from the past 12 months, average state cigarette tax from the past 12 months, and average maximum Medicaid income eligibility for pregnant women as % of FPL from the past 12 months. The regressions include county fixed effects and county-pair by year fixed effects, and are weighted using the count of each outcome at the county level. The sample size is 2.3076. Standard errors (SE) are clustered at state level and shown in paratheses. ^*^p < 0.05, ^**^p < 0.01

Table 4 shows the estimates from model (2) stratifying by birth order. Across all subgroups, there is a similar pattern of differences between models (2) and (1) to those for the full sample. All estimates of model (2) are noticeably smaller than those of model (1) and statistically non-significant. Model (2) estimates are very small and near null for firstborn and second born children. For first born children, the 95% CIs of model (2) estimates for birth weight and gestational age exclude the point estimates of model (1) for these outcomes. For second born children, the 95% CI of model (2) estimate for gestational age also rules out the estimate from model (1). For higher born infants for whom EITC amounts are largest, the estimates for birth weight, gestational age, and fetal growth rate are about one third or less of those from model (1), although their 95% CIs do not exclude the point estimates from model (1).

**Table 4:** Effects of a Ten-Percentage-Point Increase in Refundable State EITC (as % of Federal Credit) on Birth Outcomes Born to Single Low-Educated Women Aged 18–46 Years, Natality Files 1989–2018, Contiguous County Sample by Birth Order

Outcomes	EITC Effect (95% CI)	Outcome Mean
*first birth order*		
Birth weight	-0.61	3192.65
	[-5.028,3.81]	
Low birth weight	-0.0002	0.096
	[-0.002,0.002]	
Gestation weeks	0.004	38.91
	[-0.012,0.030]	
Preterm birth	0.001	0.123
	[-0.001,0.003]	
Fetal Growth	-0.021	81.82
	[-0.13,0.09]	
*second birth order*		
Birth weight (grams)	-0.85	3221.62
	[-9.54,7.85]	
Low birth weight rate (%)	-0.0002	0.091
	[-0.005,0.004]	
Gestational weeks (week)	0.0002	38.67
	[0.032,0.032]	
Preterm birth rate (%)	0.004	0.136
	[-0.004,0.005]	
Fetal growth rate (grams/week)	-0.031	83.14
	[-0.21,0.15]	
*third or higher birth order*		
Birth weight (grams)	3.50	3187.43
	[-8.18,15.17]	
Low birth weight rate (%)	-0.002	0.111
	[-0.008,0.004]	
Gestational weeks (week)	0.020	38.45
	[-0.029,0.068]	
Preterm birth rate (%)	-0.003	0.16
	[-0.009,0.003]	
Fetal growth rate (grams/week)	0.045	82.66
	[-0.17,0.26]	

Note: Each cell represents the effect of a 10%-point increase in refundable state EITCs (relative to federal credits) in tax year t-m on an outcome in birth year t in counties located in states with refundable EITC programs, compared to the border counties within each county-pair. The EITC measure, each outcome and demographic characteristics and state level control variables are the average value for each county per year. Each county may have multiple observations per year since a single county may have multiple contiguous county pairs. The model includes one EITC variable, the refundable percentage of federal credit (states with no EITC and states with non-refundable ETIC have 0 on this variable as the control group). The demographic controls include indicators for maternal age, child sex, race/ethnicity, education, and count-pair by year fixed effects. State level controls include average minimum wage (2018$) from the past 12 months, average state cigarette tax from the past 12 months, and average maximum Medicaid income eligibility for pregnant women as % of FPL from the past 12 months. The regressions are run separately for each birth order. The sample size is 69,033 for first birth order, 67,166 for second birth order, and 66,877 for third or higher birth order. The regressions include county fixed effects and county-pair by year fixed effects, and are weighted using the count of each outcome at the county level. Standard errors (SE) are clustered at state level and shown in paratheses. ^*^p < 0.05, ^**^p < 0.01

Finally estimates from model (1) for contiguous border counties only (Supplementary Tables S3 and S4) show a similar pattern of results to model (1) and even more pronounced effect estimates than those for the full sample. The 95% CIs from model (2) for effects on birth weight and fetal growth rate rule out the point estimates for those outcomes from model (1) for this sample of contiguous county borders. Taken as whole, these results suggest that the differences in estimates between model (2) comparing contiguous counties and model (1) estimated for the full sample are not due to excluding non-border counties from model (2).

## Discussion

Using data from Natality birth certificates, this paper examines the effects of refundable state EITC programs on infant health outcomes using two model. The first is a classical two-way fixed effect model that compares counties over time nationwide. The second model is an extension of the first model that focuses on changes in EITC within cross-border contiguous county pairs, which arguably accounts more for local contemporaneous economic events that may bias the estimates from the first design. From the first model, which is overall comparable to previous studies, [6, 7] we find an improvement in birth weight, gestational age, and the fetal growth rate with an increase in refundable EITC. And even though we find the largest improvement for third or higher born infants consistent with the larger EITC amount, the estimates improvements for first and second born infants whose mothers receive smaller EITC. In contrast, we find much smaller and close to null estimates from the second model for first and second born infants. For third or higher born infants, we do not observe statistically significant estimates, although the estimates are imprecise, and cannot rule out moderate to large benefits.

To interpret the magnitude of the observed regression estimates, which are intent-to-treat (average) effects of a 10%-point increase in refundable EITC, we scale the estimates by the implied income change. For third or higher born infants, the maximum benefit was around $5716 in tax year 2017. If those who qualify receive the maximum credit, the maximum income increase from a 10% increase in refundable state EITC would be about $572. Nearly 88% of single mothers of two or more children with a high school or lower education (the educational level included in this study) qualify for a credit [14]. Under this scenario, the average effect estimate would represent an effect among those who receive a credit that is 1.14 times larger. For third or higher born infants, the estimate for birth weight from model (1) suggests that a 10% increase in maximum credit translates into a 14-gram increase in birth weight (12.4 g × 1.14). For a $1,000 increase in income, this would represent about 24 g increase in birth weight. This would even be an underestimate of the implied effect as not all mothers would receive the maximum credit. Compared to implied estimates of a $1,000 income increase from the federal EITC [5] and the minimum wage, [10] which suggest a birth weight increase by about 10 and 4 g, respectively, the estimate of 24 g increase appears to be implausibly large. As noted previously, the estimate from model (2) using contiguous cross-border counties for this subgroup is not statistically significant. However, it is worth pointing that its magnitude is much smaller than that from model (1) when scaled as an income effect estimate. Specifically, the intent-to-treat estimate of model (2) for this group implies an income effect estimate of 7 gram increase with a $1000 income increase. Such an estimate is within range of these two prior estimates from the federal EITC [5] and the minimum wage [10].

Taken as a whole, our findings based on the intent-to-treat policy effect estimates and their implied income effects suggest large income effects from state refundable EITC on birth weight in classical two-way fixed effect models. When compared to previous estimates for two other income support policies, [6, 7] these estimates appear to be implausibly large. Moreover, this model suggests effects on low birth weight and gestational age for first born children whose mothers would have received little EITC. Together, these results suggest potential bias in the estimates from this model. In contrast, estimates are smaller and statistically non-significant from the second model comparing contiguous cross-border county-pairs. This second model however cannot still rule out moderate to large effects especially for third-born children because of the imprecision of estimates. They do, however, rule out some of the estimates from the first model especially for first and second born infants whose mothers receive less EITC credit. Previous research suggests improvement in health with an increase in refundable state EITC among mothers of two or more children who have high school or lower education. Some of these benefits in maternal health could translate into benefits in fetal growth and early infant health, although we are not able to statistically discern these effects when comparing contiguous cross-border counties.

Our study has limitations. This is intent-to-treat analysis, estimating average refundable EITC effects among all single mothers of high school or less, not mothers who received EITC. To address this limitation, we stratify the model by birth order as a proxy for receiving higher EITC credits. Also, the estimates from model (2) are imprecise and still do not rule out moderate to large benefits on birth weight especially for third or higher born infants, reflecting a decline in power; for this subgroup, the standard error for the estimate of birth weight in model (2) increases by about 69% compared to model (1). Finally, identifying and interpreting average treatment effects from two-way fixed effect models is complicated by issues of varying treatment time and in the case of continuous treatments such as ours also by treatment intensity differences [28]. Understanding and addressing these issues in future work of state EITC programs are important future steps.

## Conclusion

In summary, this paper adds new evidence to the literature examining the effects of refundable state EITC, on infant health. We find improvement in infant health with higher refundable state EITC in a classical two-way fixed model that compare states nationwide. However, the implied income effects from this model are large and appear to be implausible compared to estimates for the federal EITC and state minimum wage effects. In contrast, we find smaller and statistically non-significant estimates when comparing. contiguous counties across state borders.

### Electronic supplementary material

Below is the link to the electronic supplementary material.

Supplementary Material 1

## Data Availability

The data that support the findings of this study is not publicly available and researchers may submit a proposal to for access to the data from the National Center for Health Statistics (NCHS) (https://www.cdc.gov/nchs/nvss/nvss-restricted-data.htm#anchor_1553801903).

## Abbreviations

EITC: The Earned Income Tax Credits
AFDC: Aid to Families with Dependent Children
TANF: Temporary Assistance for Needy Families
NCHS: The National Center for Health statistics
