# Supplementary material for: Revisiting the effects of state earned income tax credits on infant health: a quasi-experimental study using contiguous border counties approach
Source: BMC Public Health. 2023 Dec 5;23:2422. doi: 10.1186/s12889-023-17166-6 (PMC10698941; doi:10.1186/s12889-023-17166-6)
Supplement: Supplementary file 1 — Supplementary Material 1 [file 12889_2023_17166_MOESM1_ESM.docx]

**Appendix**

Supplemental Tables and Figures

Table S1. Descriptive Statistics for Infants Born to Single Low-Educated Women Aged 18-46 Years, Natality Files 1989-2018, All County Sample

|  | Mean or % Frequency |
| --- | --- |
|  |  |
| **Birth outcomes (during past 12 months)** |  |
| Birth weight (grams) | 3209.9  (91.3) |
| Low birth weight rate (%) | 0.1  (0.03) |
| Gestational weeks (week) | 38.7  (0.4) |
| Preterm birth rate (%) | 0.1  (0.04) |
| Fetal growth rate (grams/week) | 82.8  (1.9) |
| **Demographic control variables** |  |
| Age in 5 years categories |  |
| 18-24 | 59.4 |
| 25-29 | 22.6 |
| 30-34 | 11.8 |
| 35-39 | 5.1 |
| 40-46 | 1.1 |
| Race/ethnicity |  |
| White, non-Hispanic | 39.3 |
| Black, non-Hispanic | 26.6 |
| Hispanic | 30.1 |
| Other race/ethnicity | 4.0 |
| Live birth order |  |
| first order | 38.9 |
| 2^nd^ | 29.2 |
| 3^rd^ | 17.0 |
| 4^th^ or higher | 14.9 |
| Child sex |  |
| Female | 49.0 |
| Male | 51.0 |

Note: This is county level aggregate data (one observation per county per year). The outcomes and demographic control variables are the average calculated from the individual level data. The sample size is 85925. Counts for each outcome and demographic control variables from individual level data are applies as sampling weights.

Table S2. Number of States with Refundable EITC between Tax Years 1995-2018

| **Tax year** | **States with refundable EITC programs** | | |
| --- | --- | --- | --- |
|  | **Number of states** | **Average EITC credits (% of federal credit)** | **Range of state EITC credits (% of federal credit)** |
| 1988 | 1 | 2 | 25 |
| 1989 | 2 | 3 | 30 |
| 1990 | 2 | 3 | 30 |
| 1991 | 3 | 3 | 31 |
| 1992 | 3 | 3 | 31 |
| 1993 | 3 | 3 | 31 |
| 1994 | 4 | 3 | 24 |
| 1995 | 4 | 23 | 10-33 |
| 1996 | 4 | 25 | 20-33 |
| 1997 | 5 | 22 | 10-33 |
| 1998 | 6 | 19 | 10-33 |
| 1999 | 7 | 19 | 10-33 |
| 2000 | 9 | 17 | 10-33 |
| 2001 | 9 | 20 | 10-33 |
| 2002 | 9 | 21 | 5-33 |
| 2003 | 11 | 19 | 5-33 |
| 2004 | 11 | 19 | 5-33 |
| 2005 | 12 | 18 | 5-35 |
| 2006 | 13 | 18 | 5-35 |
| 2007 | 15 | 17 | 5-35 |
| 2008 | 18 | 15 | 3.5-40 |
| 2009 | 18 | 16 | 3.5-40 |
| 2010 | 18 | 16 | 3.5-40 |
| 2011 | 19 | 17 | 3.5-40 |
| 2012 | 19 | 16 | 3.5-40 |
| 2013 | 19 | 16 | 3.5-40 |
| 2014 | 19 | 16 | 3.5-40 |
| 2015 | 20 | 16 | 3.5-40 |
| 2016 | 21 | 21 | 3.5-85 |
| 2017 | 22 | 20 | 3.5-85 |
| 2018 | 22 | 22 | 3.5-85 |

Notes: We obtained the annual data of state EITC as percentage of federal credit and refundability status for tax years 2000-2017 from <https://www.taxpolicycenter.org/statistics/state-eitc-percentage-federal-eitc>.

Number of states with refundable EITC programs include Washington, D.C.

Indiana started its state EITC in 1999 but not structured as the federal until 2003 switching to 6% of federal (so it is coded as having a refundable EITC program, but the percentage is coded as missing until 2003). See: <http://www.taxcreditsforworkersandfamilies.org/state-tax-credits/indiana/>

Washington enacted a refundable EITC in tax year 2009 at 5% of federal credit but the program was never implemented due to lack of funds. Therefore, we include Washington as a no EITC state. See: <https://www.ncsl.org/research/labor-and-employment/earned-income-tax-credits-for-working-families.aspx>

Maryland is excluded because its EITC does not follow the federal EITC structure for all years.

South Carolina and Hawaii enacted a nonrefundable EITC at 125% and 20% in tax year 2017, Montana enacted a refundable EITC at 20% in tax year 2017, but the programs were not implemented. Therefore, we include these three states as a no EITC state for the whole study period. See: <https://www.taxpolicycenter.org/statistics/state-eitc-percentage-federal-eitc>

Table S3. Effects of a Ten-Percentage-Point Increase in Refundable State EITC (as % of Federal Credit) on Birth Outcomes Born to Single Low-Educated Women Aged 18-46 Years, Natality Files 1989-2018, Border Counties Sample (Repeated Border Counties)

| Outcomes | EITC Effect  (95% CI) | Outcome Mean |
| --- | --- | --- |
| *All birth order* | | |
| Birth weight (grams) | 11.29* | 3198.72 |
|  | [2.19,20.27] |  |
| Low birth weight rate (%) | -0.005* | 0.10 |
|  | [-0.009, -0.001] |  |
| Gestational weeks (week) | 0.054* | 38.70 |
|  | [0.009,0.01] |  |
| Preterm birth rate (%) | -0.003 | 0.14 |
|  | [-0.009,0.002] |  |
| Fetal growth rate (grams/week) | 0.19* | 82.42 |
|  | [0.033,0.34] |  |

Note: Each cell represents the effect of a 10 percentage-point increase in refundable state EITCs (relative to federal credits) in tax year t-m on an outcome in birth year t. The EITC is lagged two years prior to birth year (t-2) for birth months during January to April and lagged one year prior to birth year (t-1) for birth months during May to December. estimates are county level aggregated data analyses The EITC measure, each outcome and demographic characteristics and state level control variables are the average value for each county per year. Each county may have multiple observations per year since a single county may have multiple contiguous county pairs. The model includes one EITC variable, the refundable percentage of federal credit (states with no EITC and states with non-refundable ETIC have 0 on this variable as the control group). The demographic controls include indicators for maternal age, child sex, race/ethnicity, education. State level controls include average minimum wage (2018$) from the past 12 months, average state cigarette tax from the past 12 months, and average maximum Medicaid income eligibility for pregnant women as % of FPL from the past 12 months. The sample size is 203076. The regressions include county and year fixed effects and are weighted using the count of outcomes at the county level. Standard errors (SE) are clustered at state level and shown in paratheses. ^*^ *p* < 0.05, ^**^ *p* < 0.01.

Table S4. Effects of a Ten-Percentage-Point Increase in Refundable State EITC (as % of Federal Credit) on Birth Outcomes Born to Single Low-Educated Women Aged 18-46 Years, Natality Files 1989-2018, Border Counties Sample by birth Order (Repeated Border Counties)

| Outcomes | EITC Effect  (95% CI) | Outcome Mean |
| --- | --- | --- |
| *first birth order* | | |
| Birth weight (grams) | 6.53* | 3192.65 |
|  | [1.41,11.65] |  |
| Low birth weight rate (%) | -0.003** | 0.096 |
|  | [-0.005, -0.001] |  |
| Gestational weeks (week) | 0.044* | 38.91 |
|  | [0.010,0.079] |  |
| Preterm birth rate (%) | -0.001 | 0.123 |
|  | [-0.004,0.002] |  |
| Fetal growth rate (grams/week) | 0.084 | 81.82 |
|  | [-0.001,0.17] |  |
| *second birth order* | | |
| Birth weight (grams) | 10.19* | 3221.62 |
|  | [2.28,18.10] |  |
| Low birth weight rate (%) | -0.004* | 0.091 |
|  | [-0.008, 0.0004] |  |
| Gestational weeks (week) | 0.047* | 38.67 |
|  | [0.003,0.091] |  |
| Preterm birth rate (%) | -0.003 | 0.136 |
|  | [-0.008,0.003] |  |
| Fetal growth rate (grams/week) | 0.171** | 83.14 |
|  | [0.048,0.29] |  |
| *third or higher birth order* | | |
| Birth weight (grams) | 16.93* | 3187.43 |
|  | [3.16,30.69] |  |
| Low birth weight rate (%) | -0.007* | 0.111 |
|  | [-0.013, -0.001] |  |
| Gestational weeks (week) | 0.067* | 38.45 |
|  | [0.008,0.13] |  |
| Preterm birth rate (%) | -0.006 | 0.16 |
|  | [-0.014,0.003] |  |
| Fetal growth rate (grams/week) | 0.31* | 82.66 |
|  | [0.068,0.56] |  |

Note: Each cell represents the effect of a 10 percentage-point increase in refundable state EITCs (relative to federal credits) in tax year t-m on an outcome in birth year t. The EITC is lagged two years prior to birth year (t-2) for birth months during January to April and lagged one year prior to birth year (t-1) for birth months during May to December. estimates are county level aggregated data analyses. The EITC measure, each outcome and demographic characteristics and state level control variables are the average value for each county per year. Each county may have multiple observations per year since a single county may have multiple contiguous county pairs. The model includes one EITC variable, the refundable percentage of federal credit (states with no EITC and states with non-refundable ETIC have 0 on this variable as the control group). The demographic controls include indicators for maternal age, child sex, race/ethnicity, education. State level controls include average minimum wage (2018$) from the past 12 months, average state cigarette tax from the past 12 months, and average maximum Medicaid income eligibility for pregnant women as % of FPL from the past 12 months. The regressions are run separately for each birth order. The sample size is 69033 for first birth order, 67166 for second birth order, and 66877 for third or higher birth order. The regressions include county and year fixed effects, and are weighted using the count of outcomes at the county level. Standard errors (SE) are clustered at state level and shown in paratheses. ^*^ *p* < 0.05, ^**^ *p* < 0.01.

Figure S1. Proportion of County-Pairs with Refundable EITC Differences


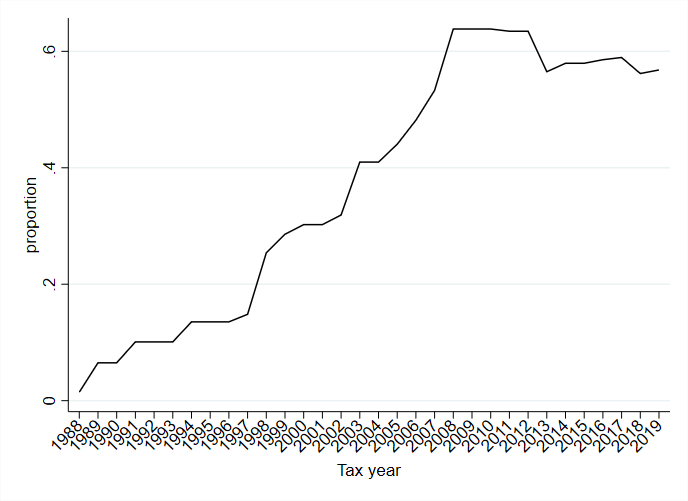


Note: state EITC data is merged with county adjacency file including all contiguous county-pairs in the United States provided by Census Bureau from NBER website (County Adjacency, May 2017).

Figure S2. Average Refundable EITC Percentage Differences Among Contiguous County-Pairs with Differences


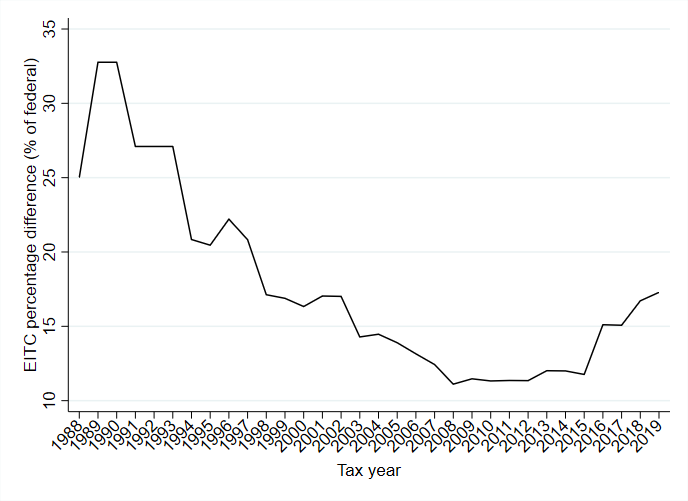


Note: state EITC data is merged with county adjacency file including all contiguous county-pairs in the United States provided by Census Bureau from NBER website (County Adjacency, May 2017)
